# The Effect of Various Parameters on a Portable Sensor for the Detection of Thin Biofilms in Water Pipes

**DOI:** 10.3390/s21134421

**Published:** 2021-06-28

**Authors:** Sachin Davis, Nathan Salowitz, Lucas Beversdorf, Marcia R. Silva

**Affiliations:** 1 Water Technology Accelerator (WaTA), University of Wisconsin-Milwaukee, Milwaukee, WI 53211, USA; davis545@uwm.edu; 2 Department of Mechanical Engineering, College of Engineering and Applied Sciences, University of Wisconsin-Milwaukee, Milwaukee, WI 53211, USA; salowitz@uwm.edu; 3 Milwaukee Water Works, Milwaukee, WI 53211, USA; lucas.beversdorf@milwaukee.gov

**Keywords:** sensor systems, system, portable sensors, non-invasive, non-destructive, biofilm

## Abstract

The use of high-frequency strain waves to perform examinations and note measurements is referred to as ultrasonic testing (UT). UT is commonly used for the detection or evaluation of flaws and characterization of materials, among other applications. A standard ultrasonic inspection system comprises a pulser/receiver, transducer, and display devices. The pulser/receiver produces electrical pulses of high voltage. The transducer generates high-frequency ultrasonic energy after being driven by the pulser. The reflected wave is then converted into an electrical signal by the transducer and is displayed on a screen. The reflected signal strength versus the time plot helps to glean information regarding the features of a defect. In this paper, we discuss the experiments performed in a laboratory setting to determine ultrasound-based biofilm sensor sensitivity in relation to changes in the surrounding environment of temperature, concentration, turbidity, and conductivity of the liquid passing through the system. The effect of the change in frequency of the sensors was also studied. The sensors being developed are small and compact, portable, can be placed on the outer walls of the desired surface, use digital signal processing techniques, and the biofilm presence on the inner walls of the surface can be monitored.

## 1. Introduction

It was van Leeuwenhoek who first described a biofilm, but the process of its formation was not fully understood until 1978. Costerton et al. [1] first described the concept that bacteria could stick to various surfaces, including a human tooth and lung, and the intestine of a cow or a rock submerged in a fast-moving stream. During Stage 1 of the growth of a biofilm, any traces of bacterial cells in the medium can be easily cleaned. However, as the bacterial cells begin to mature (i.e., at Stage 4), the cleaning process becomes both challenging and time-consuming. At this stage, more advanced cleaning methods must be used to ensure that the medium is properly cleaned and that there are no bacterial cells active. Even the smallest trace of bacterial cells can later fuse with food particles or water and can cause health hazards and increase the operation cost; moreover, the advanced cleaning process can leave traces of chemicals or radiation. The resistance of biofilms to industrial biocides is related to the limitation in mass transfer due to the matrix material [2]. The biofilm matrix limits the diffusion of sound waves only when the diffusing molecule comes into direct contact with the matrix material [3]. The biofilm phenotype is also remarkably resistant to antibacterial agents, such as antibiotics [4], and the bactericidal effects of metal ions including copper and silver. Different species of bacteria readily colonize the surface of these metals [5]. Strategies of intervention presently used for the control of biofilms in the health industry for medical devices and the water industry have the following objectives:

- Help prevent initial device contamination.
- Reduce the initial microbial cell attachment to the surface.
- Kill the biofilm-associated cells by penetrating the surface and thus removing them.

The primary mechanism that limits biofilm biomass accumulation is believed to be detachment, which refers to the passive release of biofilm particles due to mechanical or shear stress [6]. Some other common detachment mechanisms based on shear stress are grazing, abrasion, erosion, and sloughing, which helps remove biofilm particles to a certain extent [7]. Grazing usually refers to the loss of biofilm cells by the feeding activity of eukaryotic organisms, whereas abrasion is caused by collisions of biofilm cells with particles from the environment. Erosion and sloughing refer to the loss of biofilm cells by fluid frictional forces, with erosion removing small portions of the biofilm and sloughing removing intact pieces of biofilm or the entire biofilm. In contrast to detachment, dispersion is an active event in which sessile, matrix-encased biofilm cells start escaping from the biofilm, creating central voids, and leaving behind eroded biofilms [8,9,10]. Until recently, dispersion of the biofilm or the mechanism by which bacteria disperse had received little attention. Dispersion is now considered a promising avenue for biofilm control, given that it coincides with the conversion of biofilm bacteria to a planktonic mode of growth that is more vulnerable to antimicrobial agents and immune responses. As reported by numerous publications and the growing interest of pharmaceutical and start-up companies in pursuing antibiofilm products, dispersion is now a rapidly emerging field of biofilm research [11]. At the final stage of biofilm development (referred to as dispersion), sessile cells from the biofilm matrix transition to the planktonic mode of growth, which leads to a new cycle of biofilm development at new sites of colonization [12]. 

Material deterioration through corrosion, loss of heat-transfer efficiency, and mechanical clogging in fluid transport systems is one of the major issues related to biofouling. Biofouling of any materials in contact with water results in a range of adverse issues for industrial engineered water systems such as a decrease in water transport efficiency, heat exchange limitation, corrosion, and increased maintenance costs [13]. Marine biofouling is associated with an increase of at least 30% in fuel consumption by large seagoing vessels to overcome the viscous drag imposed by fouling organisms on ship hulls. An estimate of more than $US 6 billion has been reported to have been spent on repairs and preventive maintenance activities resulting from biofouling by governments and industries, highlighting the economic challenge posed by biofilms [14]. Biofilms are responsible for more than 80% of microbial infections and more than 60% of all nosocomial infections, according to the U.S. National Institutes of Health [15]. The annual incidence of biofilm-related infections is 1.96 million cases, causing an estimated 268,000 deaths, with more than $US 18 billion in direct costs spent on the treatment of these infections in the United States alone [15]. Traditional cleaning strategies sometimes involve the use of environmentally unfriendly chemicals. To reduce the impact on the environment by traditional cleaning chemicals, alternative procedures that are less severe and more eco-friendly, more respectful to material integrity, and with a lower water footprint are required [13]. In this context, ultrasonic treatments represent a promising alternative. Indeed, ultrasonic waves have been used in many environmental, industrial, and medical sectors including algal bloom control, food and beverage processing, sonochemistry, nanotechnology, mineral processing, welding, surface cleaning, medical scanning, and non-destructive testing [16]. Other applications of ultrasonic waves include the prevention of marine macro-fouling, which is usually caused by the attachment of large organisms such as barnacles, seaweed, mussels, and diatoms. This application involves devices attached to the vessel hub emitting mechanic waves in the ultrasonic (>20 kHz) frequency range [17,18,19]. Some studies have reported the inhibition of fouling in heat exchangers and pipes when applied with short pulses of high-power ultrasonic waves, for example, with pulses of 0.2 s with 100 s inter-pulse intervals per day [20], or pulses of 3 × 30 s per day [21]. Mechanisms explaining such an effect are generally not reported, but they appear to be frequency- and power-dependent, and they could be related to both mechanical effects and local cavitation phenomena [22].

Richardson invented the echo locator in 1912 based on the idea of using ultrasound for the detection of objects in water and navigation. In 1930, Langevin constructed a pulse-echo ultrasonic metal flaw detector and another ultrasonic application to detect submarines [23]. The method to determine the presence of a biofilm in its early stages used ultrasound sensors. In 1794, Spallanzani proved that bats could navigate accurately in the dark through echo reflection from the high-frequency inaudible sound. One main advantage of ultrasound is that it is non-invasive. Low-frequency ultrasounds (<5 MHz) are safe for clinical investigations, but high-frequency ultrasound waves can damage tissue [24]. The sinking of the Titanic is believed to mark the beginning of Sonar and ultrasound for medical imaging [25]. Following the Titanic tragedy in 1913, British scientist Richardson filed patents to detect icebergs using ultrasound. Chilowski and Langevin, two French scientists, started developing a device to detect submarines using ultrasound during World War I [26]. Ultrasound is widely used in industrial applications to detect defects in structures and provide biomedical imaging of cells, tissues, and organs. Ultrasound is now a valuable and flexible modality in medical imaging and often provides an additional or unique tissue characterization. Recently, ultrasound was proven to be useful in sclerotherapy, a treatment for pathological veins (or varicose veins). Critello et al. found that foams can be produced using 4% polidocanol solutions and different power levels in the case of ultrasound technique, and the foams produced were similar to those produced by the commercially available Easyfoam^®^ kit. Moreover, ultrasound generated bubbles were more stable and less prone to ageing [27]. 

An ultrasound transducer sends a transmitter pulse into a tissue and then receives an echo back. These echoes contain spatial and contrast information. This technique is like the Sonar used in marine applications. The medical ultrasound technique is, however, more complicated, since it involves the gathering of data to generate a two-dimensional grayscale image of objects which move rapidly. The reflected echoes from the tissue can be used as supportive information beyond a grayscale image to improve the analysis. Doppler ultrasound techniques can be used to detect a frequency shift in echoes and can help determine the direction in which the tissue is moving, either toward or away from the transducer. This is useful in the evaluation of structures like blood vessels or the heart (echocardiography) [28]. Ultrasound studies were used in the detection of cancellous bones by analyzing the attenuation of sound waves and the dispersion of waves in humans. A bone model which mimics the phantom and human cancellous bones was used in the study. The focus of the experiment was the analysis of physical mechanisms of ultrasonic wave propagation in a cancellous bone that governs phase velocity and attenuation coefficient as a function of frequency and porosity [29]. 

**Figure 1:**
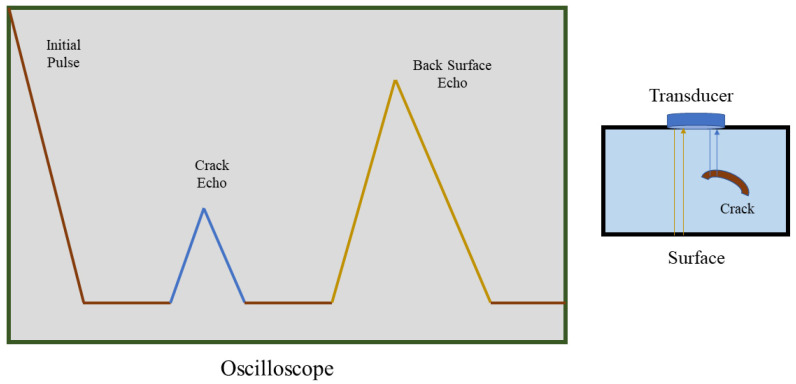
An illustration of the ultrasound detection technique using an ultrasound sensor and a surface (solid) with an internal crack. Information about the approximate size and position of the defect can be viewed using an oscilloscope.

Ultrasonic testing (UT) uses sound energy at various frequencies to analyze flaws, conduct experiments, and note measurements. UT is commonly used for the detection and evaluation of flaws and the characterization of materials, among other applications. A standard ultrasonic inspection system comprises a pulser/receiver, transducer, and display devices. The pulser/receiver produces electrical pulses of high voltage. The transducer generates high-frequency ultrasonic energy after being driven by the pulser. The reflected wave is then displayed on a screen, as shown in Figure 1, after the transducer converts it into an electrical signal. The strength of the reflected signal is displayed versus the time from when the signal generation to when the echo was received, and this information is used to determine the features of a defect [30].

It is believed that ultrasound can propagate in four different principal modes based on the way the particles vibrate or oscillate. They can propagate as longitudinal waves, shear waves, surface waves, and plate waves (especially in the case of thin metals). The most widely used propagation techniques used in ultrasonic testing are longitudinal and shear waves. With regard to longitudinal waves, the oscillations either occur in the direction of wave propagation or in the longitudinal direction. They are also called pressure waves or compressional waves, since they usually exhibit compressional and dilatational forces. Compression waves are generated in both liquids and solids because the energy undergoes a series of compressions and expansion movements as it travels through the atomic structure. If the particles oscillate transverse to the direction of propagation or at right angles, the wave is said to be a transverse or shear wave. For the effective propagation of shear waves, it requires an acoustically solid material, and therefore does not propagate well in liquids or gases. Compared to longitudinal waves, shear waves are relatively weak [31]. The ultrasonic principle is based on the reflection and refraction properties of sound. When a sound wave passes between materials of different acoustic speeds, a fraction of the wave bounces back, which is commonly referred to as reflection. The reflection phenomena are similar to that of light, where the incident wave bounces off at an angle of incidence at the boundary of the two materials. The incoming incident wave reflects off the boundary at an incident angle equal to the reflection angle similar to light. This contrasts with reflection. The acoustic impedance and refractive indices of the medium through which sound travels may not be equally dependent on the incident angle and refracted angle. The reflection coefficient describes the intensity of the reflected wave, which is equal to the intensity of the incident sound wave divided by the intensity of the reflected sound wave. The transmitted intensity is the amount of energy transmitted through the boundary, representing the non-reflected energy from the incident sound wave. A novel ultrasound sensor for the detection of biofilm inside pipes was developed and reported elsewhere [32]. 

**Figure 2:**
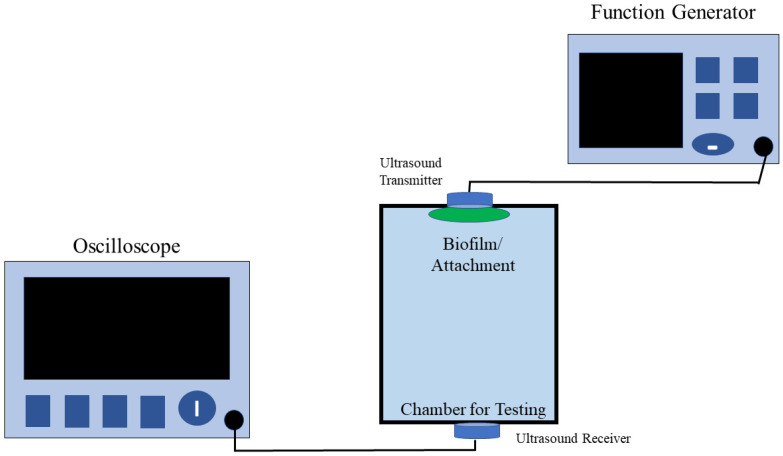
Schematic representation of the sensor arrangement. In the image, two sensors—a transmitter sensor and a receiver sensor—are placed on either side of the chamber.

Our lab has developed an ultrasound-based biofilm sensor [32] capable of detecting thin biofilms on the inner walls of a pipe or a closed surface. In our previous study, we devised a novel method for the detection of biofilms by placing the sensor in a specific position similar, to the schematic (Figure 2) shown in this paper. In that study, we were able to test the sensors on multiple surface types with different test objects. It was observed that as the thickness and type of the objects increased, the voltage output of the sensors decreased, and there was a small phase shift in the sensor output. Some of the different objects used involved were a Ziploc bag cut in half, a whole Ziploc bag, A4 paper, household aluminum foil, and common laboratory tissue paper. The experimental design involved placing two ultrasound sensors on either side of a piping system. From the previous research [33], HC-SR04 Arduino sensors were tested but proved unreliable for biofilm detection in a water medium in test chambers. While HC-SR04 sensors can be used for the measurement of the distance between objects with air as the medium, these sensors cannot be used when water is the medium and the data produced from the sensors deviate from the actual value. The sensors used in our research are consistent with output voltages and phase shifts when water is used as the medium. At the end of this research, voltage and phase shifts were detected in materials with a thickness of 40 µm or greater. The sensing technique proved efficient since the presence of any objects inside a closed system cannot be obtained using conventional techniques such as Raman microscopy, confocal laser scanning microscopy, or other microscopy methods. The new sensing technique was both non-invasive, non-destructive, and allowed in situ detection, thus eliminating the need to stop the operation of the system or the flow of media through the system. The real-time sensing also eliminates the need to wait for results from laboratory coupon testing. The novelty of the sensor is that it requires no secondary methods to validate the receiver data and requires a frequency below 20 MHz, which reduces the cost of the entire system.

In this study, we aimed to determine changes in the ultrasound sensor measurement sensitivity in relation to changes in the surrounding conditions (temperature, salt concentration, and frequency of the ultrasound sensor). The experiments performed helped understand the effect of these phenomena on the output data obtained from the sensors and the effectiveness of using the sensors in determining the presence of early-stage biofilms. The effects of turbidity and conductivity of water on sensor data were also tested. 

## 2. Materials and Methods

### 2.1. Materials

The ultrasound sensors (1ME21TR) used in this experiment have a frequency of 1 MHz (OSENON) and an operating temperature range from −20 °C to +80 °C. Their detection distance is from 0.1 m to 5 m (in liquids), and the maximum pressure they can withstand is 1.6 MPa. Other ultrasound sensors used in this experiment included the 400E10TR-1 with a frequency of 400 KHz, and 2ME30TR-1 with a frequency of 2 MHz from OSENON ultrasonics (ShenZhen, China).

A GWINSTEK AFG-2225, 2-channel, 25 MHz function generator was used to excite the ultrasound sensors and a 4-channel oscilloscope (DSOX2004A developed by Keysight technologies) was used to observe the ultrasound readings. 

Salt concentrations were varied using table salt (NaCl, Morton Iodized Salt).

Turbidity standards of 0, 10, and 20 NTU (nominal turbidity standards) were achieved by diluting 4000 NTU standards with de-ionized (DI) water. The 4000 NTU standard used was manufactured by Formazin turbidity standard Hach.

The conductivity standards used were Oakton Conductivity and TDS Standards 100 µs/cm and 1000 µs/cm.

### 2.2. Methods

The oscilloscope and function generator setup involved setting the input signal voltage from the function generator to 10 Vpp (peak-to-peak voltage) at 1 MHz. The input signal was set to a burst signal of eight cycles. The oscilloscope’s input signal was then sent to a 5 voltage/division (V/div) readout and the output signal readout for 10 V/div. We connected the probe from the output of the function generator to the first channel of the oscilloscope, and the probe for channel 1 to one ultrasound transceiver, which acted as the transmitter, and the probe from another channel of the oscilloscope (channel 4) to another ultrasound transceiver, which acted as the receiver. 

The sensitivity of the ultrasound sensors was determined by performing five different sets of experiments:

1. Change in sensitivity in relation to temperature changes.
2. Change in sensitivity in response to salt concentration changes.
3. Change in sensitivity depending on the frequency of the sensor used.
4. Effect on sensor data of changes in water turbidity.
5. Effect on sensor data of change in water conductivity.

For the change in sensitivity due to temperature change experiment, the sensors were placed on the opposite sides of a container filled with 900 mL of DI water, on which the experiments were carried out. The container was placed inside the water bath, and the temperature of the water bath was set to 20 °C. When the current temperature inside the water bath matched the setpoint temperature, the container was taken out of the water bath. The side surfaces of the container were dried out before applying the ultrasound gel to the surface of the two ultrasound transceivers, which were placed on opposite sides of the container such that they were directly opposite each other. The frequency generator was turned on and the peak-to-peak voltages on both signals were noted. The difference in time between the start of the input signal and the start of the output signal was measured to obtain the phase shift. The experiment was repeated at 30 °C, 40 °C, 50 °C, 60 °C, 70 °C, 80 °C, and 90 °C. The arrangement of the sensors on either side of the container is shown in Figure 2.

To measure the change in sensitivity with respect to changes in salt concentration, the container was filled with 900 mL of DI water at room temperature. Ultrasound gel was then applied to the ends of the ultrasound transceivers, which were placed on either side of the container such that they were on directly opposite sides to each other. The frequency generator was turned on and the peak-to-peak voltages on both signals were noted. The difference between the time at the start of the input signal and the start of the output signal was measured to identify the phase shift. Iiodized salt was placed into the container and stirred with the water to dissolve. The voltage and phase shifts were measured and noted. The experiments were repeated from 0 to 5 g of added salt in increments of 0.1 g.

To examine the change in sensitivity in relation to the frequency of the ultrasound sensor, the container was filled with 900 mL of DI water at room temperature. Ultrasound transceivers of 400 kHz were connected with probes from the oscilloscope and function generator. Ultrasound gel was applied to the transceivers’ surfaces and on either side of the container such that they were directly opposite each other. The output signal of the frequency generator was set to match the frequency rating of the transceivers. The frequency generator was turned on and the peak-to-peak voltage on both signals was measured and noted. The difference in time between the start of the input signal and the start of the output signal was measured to obtain the phase shift. The experiment was repeated with 1 MHz and 2 MHz transceivers. The 400 kHz and 2 MHz transceivers were sensors that are commercially available.

To understand the effect of water turbidity changes on sensors, the container was filled with 900 mL of water with different nominal turbidity units (NTU) standards at room temperature. Ultrasound gel was applied to the ends of the ultrasound transceivers and was placed on either side of the container such that they were on directly opposite sides. The frequency generator was turned on and the peak-to-peak voltages on both signals were noted. The difference in time between the start of the input signal and the start of the output signal was measured to identify the phase shift. The experiment was repeated at 30 °C, 40 °C, 50 °C, 60 °C, and 70 °C. The sensor arrangement on either side of the container was similar to the schematic in Figure 2. The turbidity standards used were 0 NTU, 10 NTU, and 20 NTU.

To understand the effect of changes in water conductivity on the sensors, the container was filled with 900 mL of water with different conductivity standards (µs/cm) at room temperature. Ultrasound gel was applied to the ends of the ultrasound transceivers and was placed on either side of the container such that they were on directly opposite sides. The frequency generator was turned on and the peak-to-peak voltages on both signals were noted. The difference in time between the start of the input signal and the start of the output signal was measured to identify the phase shift. The experiment was repeated at 30 °C, 40 °C, 50 °C, 60 °C, and 70 °C. The sensor arrangement on either side of the container was similar to the schematic in Figure 2. The conductivity standards used were 100 µs/cm and 1000 µs/cm. 

A beaker was filled with 900 mL of Milwaukee tap water and the conductivity and turbidity were calculated using the conductivity meter and turbidity meter, respectively.

## 3. Results

The peak-to-peak voltage and phase shift were measured at increments of 10 °C from 20 °C to 90 °C (Table 1). There was a fast increase in the voltage response from 4.8 V at 20 °C to 8.8 V at 40 °C. Then, the output voltage slowly and steadily decreased from 8.8 V at 40 °C to 8.2 V at 70 °C, before decreasing rapidly to 4.8 V at 90 °C. The highest output voltage measured was 8.8 V at 40 °C. It seems that the response voltage was affected by the temperature of the medium such that at high and low temperatures of water, the response voltage was at the lower end of measured values. The response voltage was optimal at from 40 to 70 °C.

The phase shift at different temperatures exhibited a fluctuating trend. From 20 °C to 40 °C, the phase shift decreased from 70.518 to 68.390 µs before rising to 68.844 µs at 50 °C. The phase shift then decreased further to 67.092 µs at 70 °C before rising again to 67.763 µs at 80 °C and then dropping further to 66.924 µs at 90 °C (Table 1).

The response voltage and phase shift between the input signal and response signal was measured with different concentrations of iodized salt in the water. The voltage response did not exhibit a consistent trend. From 0.0 to 0.9 g, the response voltage fluctuated between 8.6 V and 8.8 V. From 1.0 to 1.9 g the output was 9.2 V, while from 2.0 g to 2.9 g the output changed from 8.6 to 8.5 V, but the response output dropped to a low of 8.4 V from 2.5 to 2.8 g before rising back to 8.5 V at 2.9 g. From 3.0 to 3.9 g, the output fluctuated between 8.5 and 8.4 V. From 4.0 to 5.0 g, the response increased at an inconsistent rate from 8.8 to 9.2 V. The phase shift measured at those concentrations also fluctuated. However, there was a more apparent trend from 0 to 1 g. The phase shift steadily increased to a peak of 69.132 µs from 0.2 to 0.5 g before steadily dropping to 69.010 µs at 0.7 g. The phase shift then increased to 69.160 µs at 0.90 g. From 1.0 to 1.9 g the phase shift started at 68.902 µs and dropped to a low of 68.863 µs at 1.1 g. The phase shift then steadily increased to 68.924 µs from 1.1 to 1.9 g. At 2.0 g the phase shift was 68.706 µs and increased to 68.867 µs at 2.2 g. The phase shift then increased at a slower rate to 68.918 µs at 2.9 g. The phase shift at 3.0 g was 68.877 µs and then slightly increased to a peak of 68.937 µs at 3.4 g. The phase shift then dropped steadily to 68.888 µs at 3.9 g. During this period, the phase shift remained relatively constant. From 4.0 to 4.9 g, the phase shift steadily dropped from 68.912 to 68.810 µs, and there was one outlier data point of 68.930 µs at 4.5 g. The overall trend shows that the phase shift between the input signal and response signal decreases as the concentration of iodized salt increases (Table 2). 

The ratio of the output voltage with respect to the input voltage (Vout/Vin) and the phase shift between the input signal and the response signal was measured at three different input signal frequencies: 400 kHz, 1 MHz, and 2 MHz. At the lower frequency of 400 kHz, the ratio was at its lowest (0.18), while at the higher frequency of 2 MHz, the ratio was similarly low at 0.16. At 1 MHz, the ratio was significantly higher at 0.55, indicating that 1 MHz is the optimal frequency of the three tested. The phase shift increased with the frequency. The phase shift of −0.028 µs at 400 kHz was similar to the phase shift of −0.074 µs at 1 MHz. By comparison, the phase shift of 0.176 µs at 2 MHz was significantly higher (Table 3).

**Figure 3:**
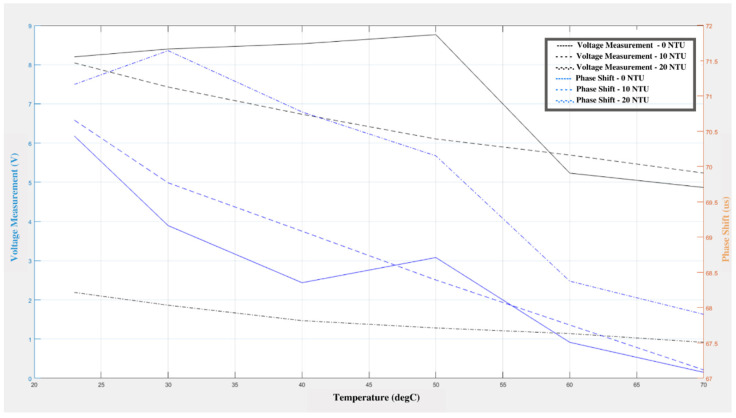
Variation in voltage and phase shift in relation to changes in the turbidity and temperature of the water.

The response of the sensor to changes in water turbidity can be seen in Figure 3. As the water temperature changes, it can be observed that the voltage and phase shift decrease gradually. Even though there was a gradual rise in the voltage and phase shifts for the 0 NTU standard, this trend cannot be seen in the other cases. The decrease in the voltage and phase shift may be related to the change in temperature, since the same effect can be observed in the results of the temperature change experiment. This means that the change in turbidity does not have a substantial adverse effect on the sensitivity of the sensor. The maximum deviation in the turbidity experiment was 0.3 V for the output and 0.288 µs for the phase shift measurement. The minimum deviation was 0.09 V for the output measurement and 0.063 µs for the phase shift measurement.

**Figure 4:**
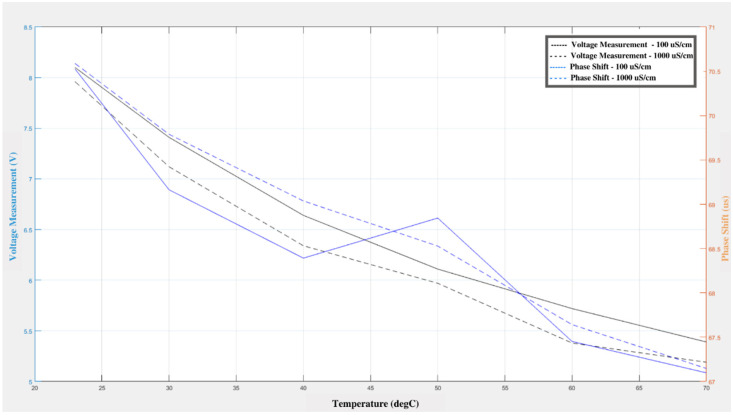
Variation in voltage and phase shift in response to changes in the conductivity and temperature of the water.

The response of the sensor to changes in water conductivity can be seen in Figure 4, which shows the voltage and phase shift changes in relation to the conductivity and temperature of the water. As the water temperature changes, it can be observed that the voltage and phase shift decrease gradually. Even though there was a gradual rise in phase shift for the 100 µs/cm conductivity standard, this trend is not seen in the other case. The decrease in the voltage and phase shifts can be related to the change in temperature since the same effect can be observed in the temperature experiment’s change. This means that the change in conductivity does not have a substantial adverse effect on the sensors’ sensitivity. The maximum deviation for the turbidity experiment was 0.34 V for the output measurement and 0.626 µs for the phase shift measurement, while the minimum deviation was 0.05 V for the output measurement and 0.047 µs for the phase shift measurement.

Milwaukee drinking water was measured as 0.02 NTU, while the conductivity was approximately 300 µs/cm. Therefore, all of the experiments were performed in the normal range of drinking water and the findings can be applied to potable water systems.

## 4. Discussion

The advantages of using the ultrasound sensors [34] are as follows:

1. The color or transparency has no effect on the sensor’s reading, since ultrasonic sensors reflect sound off objects, so they are not affected by the color or transparency of objects.
2. Dark environments have no effect on an ultrasonic sensor’s detection ability, unlike proximity sensors using light or cameras.
3. They are not heavily affected by dust, dirt, or high moisture environments.
4. They have high accuracy in measuring thickness and distance to the parallel surface compared to many other methods.
5. Their high frequency, sensitivity, and penetrating power make it easy to detect external or deep objects.
6. They are easy to use and not dangerous during operation to nearby objects, people, or equipment.

However, several factors can impact the operation of the sensors, and below we discuss some factors based on the results of this research. Temperature fluctuations impact the speed of the sound waves produced by ultrasonic sensors. It may seem that a target is closed even when the target has not moved when the temperature increases, because sound waves will travel faster to and from the target. The flow of air due to fans or other pneumatic equipment also deflect or disturb the path of the ultrasonic wave. This could lead to the sensor failing to recognize the correct location of a target [35]. In their experiment to study the effects of the environment temperature on the characteristic of the ultrasonic sensor, Stanescu et al. concluded that a rise in temperature implies an increase of the error when measuring with the sensor [36]. In our study, the phase shift decreased with an increase in temperature. The output voltage was lower at temperatures below 30 °C and higher than 80 °C when compared to the output voltage at temperatures between 30 and 80 °C, indicating that the best operation at those temperatures. Compared to the HC-SR04 Arduino sensors, which are most used in other applications, our sensors reported here are less sensitive to temperature fluctuations. The HC-SR04 sensors are more sensitive to temperature changes since the range of detection of the sensors is low, and temperature effects on the speed of sound produce errors in measurement. 

The voltage output (Figure 3) observed on the receiver sensor decreased with an increase in turbidity of the solution. Similarly, there was a significant change in phase shift as the turbidity of the medium increased. This trend is similar to when biofilm is present in the system because biofilm tends to increase the turbidity of the medium. Turbidity is often considered a measure of cell density when measuring a culture sample. Microbiologists also use machines called photometers and spectrophotometers that shine several types of light through culture samples to determine turbidity. The general conclusion is that as turbidity increases so does the number of cells within the culture [37]. Douterelo et al. observed significant positive correlations between turbidity and the relative abundance of several bacterial groups. They also observed that the turbidity patterns in response to each repeated flushing operation were consistent for both cast iron and plastic pipes across their length over time. This suggested continuous and repeatable processes across the length of the pipe, and similar strengths of the biofilm accumulation in response to the increased flushing forces [38].

The voltage output (Figure 4) observed on the receiver sensor decreased with increasing conductivity of the solution. Similarly, there was a significant change in phase shift as the conductivity of the medium increased. This is similar to the trend when biofilm is present in the system since biofilm tends to increase the conductivity of the medium. There have been striking results that indicate the increase in conductivity even before the multiplication of bacteria takes place. This has been proven in studies that measured bacteria count and electrical conductivity in a solution containing small inoculum of back *subtilis*, glucose, and peptone. The estimation of the amino acids formed in the medium revealed a significant correlation between the concentration of bacteria and conductivity of the suspension at various stages of bacterial growth. The results suggested that with the increase in bacterial formation in the medium, the ratio of amino-acid formation increased. This provides evidence for the view that substances are passed from the cells into the medium during the lag phase, which is supported by our finding that the conductivity of a suspension of washed bacteria in water with high conductivity increases exponentially over time [39].

The ultrasound sensors that we used in this study are highly economical and easy to operate compared to most other sensors available on the market. The scope for future related research includes the characterization and identification of the thickness of biofilms using digital signal processing techniques along with computerized algorithms to increase the accuracy of the measurements. The use of an array of sensors will also be tested, in which multiple sensors will be placed on the pipe loop to pinpoint the exact location of the biofilm and its thickness on a long pipe loop. Other research activities include testing the sensor system in a real-world environment, i.e., testing the sensors on a pipe loop in a real-world scenario to understand how the sensors react to real-world conditions and make any necessary modifications. There are plans to incorporate machine learning to train the sensors to recognize and characterize the thickness and type of deposit on the inner walls of the pipe to make the calculations more accurate. The sensors will then be coupled with a microprocessor system such as the Raspberry Pi to include digital signal processing and machine learning algorithms. It is our aim for it to be possible for sensor data to be sent to a handheld user interface system which will give an indication of the presence of a biofilm to the engineer or technician. This system will be developed into a commercial product for industries looking to measure biofilms in their piping systems.

## 5. Conclusions

The phase shift difference between the experiment sets was negligible from the change in concentration experiment described in this paper (around 0.5 µs). This suggests that the sensor output does not vary with the change in dissolved materials inside the container. From the change in temperature experiment, it was found that there is a slight effect on the sensor output as the temperature changes. From the change in frequency experiment, it was evident that a 1 MHz ultrasound sensor is more suitable for the application because it gives more voltage range for data analysis compared to the other two sensors we tested. Our experiments showed that the 1 MHz ultrasound sensor can be used to detect biofilms at an early stage (i.e., Stage I).

## Figures and Tables

**Table 1:** Results of the effects of temperature change study.

Temperature (°C)	Phase Shift (µs)	Output Voltage (Vpp)
20	70.518	4.8
30	69.158	5.2
40	68.390	8.8
50	68.844	8.5
60	67.439	8.4
70	67.092	8.2
80	67.763	7.6
90	66.924	4.8

**Table 2:** Results of the effects of change in dissolved salt concentrations.

Weight of Salt Dissolved (g)	Phase Shift (µs)	Output Voltage (Vpp)
0.0	69.052	8.8
0.5	69.132	8.8
1.0	68.902	9.2
1.5	68.904	9.2
2.0	68.706	8.6
2.5	68.857	8.4
3.0	68.877	8.5
3.5	68.780	8.5
4.0	68.912	8.8
4.5	68.930	9.2
5.0	68.810	9.2

**Table 3:** Results of the effects of change in frequency on the sensor.

Frequency (MHz)	Phase Shift (µs)	Voltage Ratio (Vout/Vin)
0.4	−0.028	0.18
1.0	−0.074	0.55
2.0	0.176	0.16

## Data Availability

The authors confirm that the data supporting the findings of this study are available within the article.
